# Protocol for surveillance of antimicrobial-resistant bacteria causing community-acquired urinary tract infections in low-income countries

**DOI:** 10.1371/journal.pone.0304388

**Published:** 2024-05-31

**Authors:** Mtebe Venance Majigo, Stephen Mshana, Erick Komba, Nyambura Moremi, Mecky Matee

**Affiliations:** 1 SACIDS Foundation for One Health, Dar es Salaam, Tanzania; 2 Muhimbili University of Health and Allied Sciences, Dar es Salaam, Tanzania; 3 Catholic University of Health and Allied Sciences, Dar es Salaam, Tanzania; 4 Sokoine University of Agriculture, Morogoro, Tanzania; 5 National Health Laboratory Quality Assurance and Training Centre, Dar es Salaam, Tanzania; North Carolina State University, UNITED STATES

## Abstract

The spread of drug-resistant bacteria into the community is an urgent threat. In most low-middle-income countries (LMICs) settings, community-acquired infection (CAI) is empirically treated with no data to support the choice of antibiotics, hence contributing to resistance development. Continuous antimicrobial resistance (AMR) data on community-acquired pathogens are needed to draft empirical treatment guidelines, especially for areas with limited culture and susceptibility testing. Despite the importance of addressing antibiotic-resistant pathogens in the community setting, protocols for the surveillance of AMR bacterial infections are lacking in most (LMICs). We present a protocol for surveillance of AMR in LMICs using urinary tract infection (UTI) as a proxy for CAI to enable users to quantify and establish the drivers of AMR bacteria causing UTI. The protocol intends to assist users in designing a sustainable surveillance program for AMR in the community involving children above two years of age and adults presenting to a primary health facility for healthcare. Implementation of the protocol requires initial preparation of the laboratories to be involved, surveillance areas, selection of priority bacteria and antimicrobials to be used, and the design of a coordinated sampling plan. Recruitment should occur continuously in selected health facilities for at least 12 months to observe seasonal trends of AMR. At least 10 mL of clean-catch mid-stream urine must be collected into 20 mL calibrated sterile screw-capped universal bottles lined with 0.2 mg boric acid and transported to the testing laboratory. Utilise the data system that generates standard reports for patient care to be shared internally and externally in the regions and the world through global platforms such as the Global Antimicrobial Resistance Surveillance System.

## Introduction

Antimicrobial resistance (AMR) is a global public health crisis that impacts livelihoods, food security, and development, particularly in low-middle-income countries (LMIC). The spread of drug-resistant bacteria into the community is an urgent threat associated with increased morbidity, mortality, healthcare costs, and antibiotic use [1–3]. Among drug-resistant pathogens, extended-spectrum β-lactamase producing Enterobacterales (ESBL-PE) and multidrug-resistant to non-β-lactam antibiotics are currently the most critical threats to public health [4–6]. ESBL-PE strains are clinically significant because they limit the effectiveness of cephalosporins, one of the most commonly used antibiotics [7]. Delay recognition and inappropriate treatment of infections caused by multidrug-resistant (MDR) pathogens, such as ESBL-PE, have increased mortality [2, 8].

Urinary tract infection (UTI) leads to significant morbidity and health care expenditures, particularly in LMIC [9]. The predominant pathogens causing UTI are gram-negative bacteria from the Enterobacterales group. These pathogens include Uropathogenic *E*. *coli*, *Klebsiella pneumoniae* complex, *Proteus* spp., and *Enterobacter* spp [10]. The World Health Organization (WHO) has classified Enterobacterales that are resistant to carbapenems and third-generation cephalosporins as critical priority pathogens. This classification necessitates the development of research and development strategies for new antibiotics [10].

In most LMIC settings, UTI is empirically treated with no data to support the choice of antibiotics, hence contributing to resistance development. Continuous AMR data on community-acquired pathogens are needed to draft empirical treatment guidelines, especially for areas with limited culture and susceptibility testing [11]. With such data, policymakers can design appropriate AMR control strategies that help guide community practitioners. [12] Surveillance data on UTI can assist in understanding community-level magnitude, drivers, and transmission dynamics.

Despite the importance of addressing antibiotic-resistant pathogens in the community setting, protocols for the surveillance of AMR bacterial infections are lacking in most LMICs [13, 14]. Access to data about AMR drivers in community settings is necessary for governments and scientists to make evidence-based policy and intervention decisions. The protocol is required to assist users in designing a sustainable surveillance program for AMR and Community-Acquired Infections (CAI) [12].

We present a protocol for surveillance of AMR in low-income countries using UTI as a proxy for CAI to enable users to quantify and establish the drivers of community-acquired AMR bacteria causing UTI. We conducted a cross-sectional health center-based survey as a pilot for the protocol. The survey was conducted in Tanzania, Mwanza, and Dar es Salaam regions for five months from July to November 2021. The findings from the survey provide evidence that the protocol works [15, 16]. In addition, the protocol aligns with WHO AMR surveillance guidelines [17].

## Materials and methods

The material and methods for implementing the protocol for surveillance of antimicrobial-resistant bacteria causing community-acquired urinary tract infections in low-middle-income countries are published on protocols.io. https://protocols.io/view/survsilence-of-antimicrobial-resistant-bacteria-c-c7ryzm7w. The protocol is included for printing as a supporting information file (S1 File) with this article.

## Ethics declarations

The National Institute for Medical Research (NIMR) cleared the study with a certificate numbered NIMR/HQ/R.8a/ Vol.IX/3580 of 16 December 2020, which resulted in the development of this protocol. In implementing this protocol, ethics approval must be sought according to national guidelines; confidentiality of patients’ data should be ensured.

## Expected results

Implementation of this protocol is expected to give the following results: 1) Strengthen the existing AMR surveillance system, including epidemiological skills (sample design, data analysis, and reporting), sample collection and processing, laboratory diagnostic capacity, and data management and analysis. 2) Generate baseline estimates of the prevalence of AMR bacteria causing community-acquired UTI, of which future surveillance rounds can be compared to identify new strains, patterns, and trends in prevalence and dispersion. 3) Document the diversity of phenotypes of uropathogens based on the susceptibility patterns in the community. 4) Identify the primary drivers of community-acquired AMR pathogens causing UTI. 5) Inform priorities and evolution of the AMR surveillance system to improve understanding of the prevalence and consequences of AMR in the community.

The protocol is designed to be adopted by local governments and integrated into existing national AMR surveillance systems. Ongoing AMR surveillance is crucial for tracking resistance patterns. This surveillance provides essential data to inform policy and decision-making, helping to mitigate the consequences of identified AMR.

This protocol outlines how to conduct alert organism tracking for MDR bacteria causing community-acquired UTIs and is complementary to routine surveillance. Alert organism tracking is generally not the first step of a surveillance system but entails the addition and prioritization of particular pathogens of public health significance. Alerts will automatically be generated by the WHONET software following data entry due to pre-defined algorithms fed into the software.

## Supporting information

S1 File Step-by-step protocol for conducting surveillance of antimicrobial-resistant bacteria causing community-acquired urinary tract infections. The protocol is also available on protocols.io: dx.doi.org/10.17504/protocols.io.kqdg3xdneg25/.
(PDF)

## References

[1] Hu B, Ye H, Xu Y, Ni Y, Hu Y, Yu Y, et al. Clinical and economic outcomes associated with community-acquired intra-abdominal infections caused by extended spectrum beta-lactamase (ESBL) producing bacteria in China. Current Medical Research and Opinion. 2010;26(6):1443–9. doi: 10.1185/03007991003769068 20394469

[2] Pitout JD, Laupland KB. Extended-spectrum β-lactamase-producing Enterobacteriaceae: an emerging public-health concern. The Lancet infectious diseases. 2008;8(3):159–66. 18291338 10.1016/S1473-3099(08)70041-0

[3] Topaloglu R, Er I, Dogan BG, Bilginer Y, Ozaltin F, Besbas N, et al. Risk factors in community-acquired urinary tract infections caused by ESBL-producing bacteria in children. Pediatric nephrology. 2010;25:919–25. doi: 10.1007/s00467-009-1431-3 20151161

[4] Dhillon RH-P, Clark J. ESBLs: a clear and present danger? Critical care research and practice. 2012;2012. doi: 10.1155/2012/625170 21766013 PMC3135063

[5] Seni J, Najjuka CF, Kateete DP, Makobore P, Joloba ML, Kajumbula H, et al. Antimicrobial resistance in hospitalized surgical patients: a silently emerging public health concern in Uganda. BMC research notes. 2013;6:1–7. 23890206 10.1186/1756-0500-6-298 PMC3729663

[6] Vanitha A. WHO global priority pathogens list on antibiotic resistance: an urgent need for action to integrate One Health data. Perspectives in Public Health. 2018;138(2):87–8. doi: 10.1177/1757913917743881 29465015

[7] Shah AA, Hasan F, Ahmed S, Hameed A. Characteristics, epidemiology and clinical importance of emerging strains of Gram-negative bacilli producing extended-spectrum β-lactamases. Research in microbiology. 2004;155(6):409–21. 15249058 10.1016/j.resmic.2004.02.009

[8] Wani K, Thakur M, Fayaz AS, Fomdia B, Gulnaz B, Maroof P. Extended spectrum B-lactamase mediated resistance in Escherichia coli in a tertiary care hospital. International journal of health sciences. 2009;3(2):155. 21475532 PMC3068822

[9] Shiralizadeh S, Taghizadeh S, Asgharzadeh M, Shokouhi B, Gholizadeh P, Rahbar M, et al. Urinary tract infections: raising problem in developing countries. Reviews and Research in Medical Microbiology. 2018;29(4):159–65.

[10] Organization WH. Prioritization of pathogens to guide discovery, research and development of new antibiotics for drug-resistant bacterial infections, including tuberculosis. World Health Organization, 2017 9240026436.

[11] Klinker KP, Hidayat LK, DeRyke CA, DePestel DD, Motyl M, Bauer KA. Antimicrobial stewardship and antibiograms: importance of moving beyond traditional antibiograms. Therapeutic Advances in Infectious Disease. 2021;8:20499361211011373. doi: 10.1177/20499361211011373 33996074 PMC8111534

[12] Pezzani MD, Mazzaferri F, Compri M, Galia L, Mutters NT, Kahlmeter G, et al. Linking antimicrobial resistance surveillance to antibiotic policy in healthcare settings: the COMBACTE-Magnet EPI-Net COACH project. Journal of Antimicrobial Chemotherapy. 2020;75(Supplement_2):ii2–ii19. doi: 10.1093/jac/dkaa425 33280049 PMC7719409

[13] Frost I, Kapoor G, Craig J, Liu D, Laxminarayan R. Status, challenges and gaps in antimicrobial resistance surveillance around the world. Journal of Global Antimicrobial Resistance. 2021;25:222–6. doi: 10.1016/j.jgar.2021.03.016 33845163

[14] Iskandar K, Molinier L, Hallit S, Sartelli M, Hardcastle TC, Haque M, et al. Surveillance of antimicrobial resistance in low-and middle-income countries: a scattered picture. Antimicrobial Resistance & Infection Control. 2021;10:1–19. doi: 10.1186/s13756-021-00931-w 33789754 PMC8011122

[15] Masoud SS, Majigo M, Silago V, Kunambi P, Nyawale H, Moremi N, et al. Utility of dipstick urinalysis in the diagnosis of urinary tract infections among outpatients in Mwanza and Dar es Salaam regions in Tanzania. Bulletin of the National Research Centre. 2024;48(1):6.

[16] Silago V, Moremi N, Mtebe M, Komba E, Masoud S, Mgaya FX, et al. Multidrug-resistant uropathogens causing community acquired urinary tract infections among patients attending health facilities in Mwanza and Dar es Salaam, Tanzania. Antibiotics. 2022;11(12):1718. doi: 10.3390/antibiotics11121718 36551375 PMC9774515

[17] Who. Global antimicrobial resistance surveillance system: manual for early implementation. Who Geneva, Switzerland; 2015.

