# Supplementary material for: Protocol for surveillance of antimicrobial-resistant bacteria causing community-acquired urinary tract infections in low-income countries
Source: PLoS One. 2024 May 31;19(5):e0304388. doi: 10.1371/journal.pone.0304388 (PMC11142477; doi:10.1371/journal.pone.0304388)
Supplement: S1 File — The protocol is also available on protocols.io: dx.doi.org/10.17504/protocols.io.kqdg3xdneg25/. (PDF) [file pone.0304388.s001.pdf]

ddxd27i6

surveillance of antimicrobial-resistant bacteria causing community-acquired urinary tract infections in low-income countries V.(ddxd27i6)

Mtebe Venance Majigo[1], Stephen Mshana[2], Erick Komba[3], Nyambura Moremi[4], Mecky Matee[1]

[1]Muhimbili University of Health and Allied Sciences; [2]Catholic University of Health and Allied Sciences; [3]Sokoine University of Agriculture; [4]National Health Laboratory Quality Assurance and Training Centre

Mtebe Venance Majigo
Muhimbili University of Health and Allied Sciences

Protocol Info: Mtebe Venance Majigo, Stephen Mshana, Erick Komba, Nyambura Moremi, Mecky Matee . surveillance of antimicrobial-resistant bacteria causing community-acquired urinary tract infections in low-income countries. **protocols.io**
https://protocols.io/view/surveillance-of-antimicrobial-resistant-bacteria-c-ddxd27i6

Created: April 21, 2024

Last Modified: May 17, 2024

Protocol Integer ID: 100037

Keywords: Surveillance, urinary tract infection, antimicrobial resistance, community-acquired infection

Funders Acknowledgement:
Fleming Fund Regional Grant
Grant ID: FF49/440

Abstract

The protocol intends to assist users in designing a sustainable surveillance program for AMR in the community involving children above two years of age and adults passively presenting to a lower health facility for healthcare. Implementation of the protocol requires initial preparation of the laboratories to be involved, surveillance areas, selection of priority bacteria and antimicrobials to be used and the design of a coordinated sampling plan. Recruitment should occur continuously in selected health facilities for at least 12 months to observe seasonal trends. At least 10 mL of clean-catch mid-stream urine must be collected into 20 mL calibrated sterile screw-capped universal bottles lined with 0.2 mg boric acid and transported to the testing laboratory. Utilize the data system that generates standard reports for patient care and to be shared internally and externally in the regions and the world through global platforms such as the Global Antimicrobial Resistance Surveillance System.

Guidelines

1. Decide on the target population and enrollment criteria
2. Selection of the target laboratories, surveillance areas, bacteria, and antimicrobials to be involved in the surveillance
3. Prepare a sampling plan and sample size
4. Outline the roles of different laboratories involved in the surveillance and set agreement
5. Laboratory procedures for the generation of antimicrobial resistance data
6. Proficiency testing and internal quality assurance
7. Process for data management, analysis, reporting and sharing

Materials

PDF Annex 1- Data collection form.pdf 106KB

PDF Annex 2- Standard Operating Proced… 120KB

Before start

PREPARATIONS BEFORE BEGINNING AMR SURVEILLANCE

The initial preparation is necessary to implement this protocol for AMR surveillance effectively; consideration should be taken for the following areas.

1) ***Laboratories, Surveillance Areas, Bacteria, and Antimicrobials*:** Identify health facilities to enroll in the surveillance, identify appropriate laboratories for microbiologic analysis near data collection sites, agree on specific bacteria to be included in surveillance, and identify antimicrobials for susceptibility testing according to local conditions and global standards.

2) ***Sample collection:*** Design a coordinated sampling plan and sample collection form, prepare standard operating procedures (SOPs) for collection and transportation, prepare sample collection kits, trial collection of samples in each surveillance area, and prepare a sample correction timetable.

3) ***Laboratory preparation:*** Define necessary capacities for different levels of laboratories for participation; select laboratories that meet criteria and development agreements. Develop SOPs for culture and identification, antimicrobial susceptibility testing (AST), quality assurance, and data management. Train laboratory staff on surveillance-related procedures and testing and trial laboratory procedures for a few samples at each laboratory.

4) ***Stakeholders and community engagement***: Engage key stakeholders from public, private, and non-profit organisations and political and community leaders to define target populations and surveillance sites and help inform communities. Develop agreements with enrolled health facilities for participation; develop an agreement for AMR data sharing and isolate transfer.

1 **TARGET POPULATION AND ENROLMENT CRITERIA**

Sampling needs to involve children above two years of age and adults (pregnant and non-pregnant women and men) who are residents of a given surveillance area presenting to a health facility for health care within that same surveillance area to ensure linkage of surveillance data to a particular community. Patients with signs and symptoms of UTI less than 48 hours after hospitalisation or as an outpatient without healthcare-associated risk factors (hemodialysis, previous hospitalisation within 30 days, or receiving care in a nursing home) will be eligible for enrolment [1]. Recruitment should occur continuously in selected health facilities for at least 12 months to observe sessional trends of AMR. Clinical symptoms, signs, and history should be used to consider a presumptive UTI case. In addition, the presumptive UTI should be categorised into uncomplicated, complicated, and catheter-associated [2], as indicated in Table 1.

Table 1: Clinical Classification symptoms and signs of UTI

A	B
**Category**	**Clinical Description**
Uncomplicated UTI (cystitis)	Offensive smelling urine, painful urination, frequency of urination, urgency of urination, cloudy urine, positive urine dipstick test (leucocytes, nitrites, blood), positive urine microscopy (leucocytes, RBCs, bacteria)
Complicated UTI (Pyelonephritis, Uropsepsis)	Fever, Rigors, Flank pain, Painful urination, Cloudy urine, Costovertebral angle tenderness, Urine microscopy – (leucocytes, RBCs, bacteria)
Catheter associated (CA)-UTI	Presence of indwelling urinary catheters for two days or more with signs and symptoms of UTI and no other source of infection. Presence of ≥ 10Λ3 CFU/mL in a single catheter urine specimen or in midstream urine, despite removal of urinary catheter in the previous 48hr

2

TARGET LABORATORIES, SURVEILLANCE AREAS, BACTERIA, AND ANTIMICROBIALS

1. Laboratories and Surveillance Areas

The areas where samples will be collected are referred to as surveillance areas. Each surveillance area should bear the following attributes: primary health facility (dispensary, health centre, district hospital); be within 50 km of a laboratory capable of performing culture and sensitivity as per national standards; selected sites should represent geographic coverage and diversity of the country.

2. Target Bacteria

The target bacteria must include all potential uropathogens for community-acquired UTI in the surveillance area. Table 2 contains the list of uropathogens commonly reported [1]. Most gram-negative bacteria causing UTI can produce ESBL, other enzymes that confer resistance to carbapenem, and third and fourth-generation cephalosporins. WHO classified these common pathogens as critical priorities [3]. Data on WHO-priority pathogens are needed to advance understanding of their emergence and spread. *Escherichia coli,* the commonest pathogen isolated from urine, is an important zoonotic pathogen in humans;

they are commensal organisms both in humans and animals and may act as an indicator for resistance patterns associated with gram-negative organisms in communities with limited capacity for culture and sensitivity.

Table 2: List of Uropathogens commonly causing UTI

A
**Gram-Negative Bacteria**
1. Enterobacterales: *Escherichia coli, Klebsiella pneumoniae, Proteus mirabilis, Enterobacter* spp, *Morganella morganii, Providencia stuartii, 2. Pseudomonas* spp, *3.* Other non-fermenting gram-negative rods
**Gram-Positive Bacteria**
1. Staphylococcus saprophyticus 2. Enterococcus spp, *3. Staphylococcus aureus, 4. Streptococcus agalactiae*

2.1 Selection of Bacteria and Diagnostic Tests for Individual Laboratory

Laboratories participating in antimicrobial resistance (AMR) surveillance may be unable to culture and conduct antimicrobial susceptibility testing (AST). The laboratory procedures carried out at each laboratory should match their capacity. For example, some laboratories may test for all pathogens, while others may test for only one initially. If the selected laboratory has limited capacity, it may perform culture, identify target bacterial species, and transfer isolates to the national or zonal level for AST.

The recommended order of priority for building capacity to culture, identify, and conduct AST for uropathogens: 1) *E. coli* is the priority bacteria for strengthening diagnostic capability in surveillance laboratories. As the most common cause of UTI, implementing this protocol, *E. coli* should be a priority. 2) Other common pathogens in the family Enterobacterales include *Klebsiella pneumoniae, Enterobacter cloacae* complex, *Proteus* spp. etc. 3) Gram-positive uropathogens such as *Staphylococcus saprophyticus,* Enterococcus spp., *Staphylococcus aureus*, *Streptococcus agalactiae,* and other Coagulase-negative staphylococci which have been identified to be uropathogens.

3. Target antimicrobials

This protocol is designed to contribute to understanding the magnitude and drivers of community-acquired UTI due to AMR bacteria. The panel of antimicrobial agents for AST should be selected from the critically important antimicrobial classes based on WHO recommendations and used to treat UTI. Selected pathogens should be tested for susceptibility to the antibiotics listed in Table 3 as an example based on the Clinical Laboratory Standard Institute (CLSI) or the European Committee on Antimicrobial Susceptibility Testing (EUCAST) list for uropathogens.

Table 3: The list of isolate-antimicrobial pairs for antimicrobial susceptibility testing

**Isolate**	**Antimicrobial for AST**		
1st line	2nd line	3rd line	
Enterobacterales	*Ampicillin, Amoxicillin-Clavulanic acid, Ciprofloxacin, Nitrofurantoin, Trimethoprim-Sulfamethoxazole*	*Gentamicin, ceftriaxone, Cefotaxime, Ceftazidime,*	*Cefepime, Amikacin, Piperacillin-tazobactam, Tigecycline,*

			Meropenem, Impenem, colistin
Non-fermenting Gram negative	*Piperacillin, Gentamicin, Ciprofloxacin, Nitrofurantoin*	*Ceftazidime, Piperacillin-tazobactam, Cefepime*	*Amikacin, Tigecycline, Meropenem, Impenem, colistin*
Gram positive uropathogens	*Penicillin, Erythromycin, ciplofloxacin, Nitrofurantoin, Trimethoprim-Sulfamethoxazole*	*Linezolid, Clindamycin, Amikacin, Tigecycline, Vancomycin,*	

3 **SAMPLING PLAN**

Once the surveillance areas (health facilities, catchment areas, and nearby laboratories), target bacteria, and antimicrobial agents have been identified, the next step is to design a sampling plan. The sampling plan should centre on diagnosing UTI cases at participating health facilities, fulfilling the definition of community-acquired UTI. The sampling plan should include the number of samples collected nationally and within each surveillance area. Additionally, the sampling plan should describe the justification for the number of samples to be collected, the types of samples, methods of sample collection, and a sampling timetable. Also, the plan should show the steps for sample collection, storage, transportation, and the number of hours allowable at each stage, given different conditions. Since the protocol proposes surveillance of routine patient care, the total number of samples to be collected depends on the duration of surveillance and the type of health facilities involved.

1. Sampling Timetable

The sampling timetable should consider that specimens should reach the laboratory during working hours. Ideally, if the patient attends an outpatient clinic, they should provide the sample before leaving. Depending on the type of facility, the sampling will be as patients visit the hospital during clinic days. The number of samples will depend on the volume of patients presenting with signs and symptoms of UTI in selected health facilities.

2. Urine Sample Collection and Handling

At least 10 mL of clean-catch mid-stream urine (MSU) must be collected into 20 mL calibrated sterile screw-capped universal bottles lined with 0.2 mg boric acid. Boric acid reduces the rate of bacterial multiplication, which could affect the colony-forming unit (CFU) determination. The collection time should consider staff availability and the distance to the testing laboratory. In all facilities, samples should be collected between 0900 and 1200 hours. A health worker should instruct adult participants on aseptic techniques during collection and ensure the appropriate container is given to the patient. A health worker should assist parents in collecting urine specimens from children. Children above two years can be instructed and assisted by parents/guardians to collect MSU. Catheter and supra-pubic urine can be collected, processed, and interpreted per the approved standard operating procedure (SOP) in the respective laboratory. However, the urine collection and transportation SOP needs to be developed per national guidelines.

A health worker should label each specimen container with the unique identifier, location, time, and date of specimen collection. Ensure that the identification number on the form matches the ID number on the urine container.

3. Sample transportation

All preserved specimens must be transported to the participating laboratory for analysis within eight hours. In case of delay, the specimen should be refrigerated 2-8°C and processed within 24hrs. Upon arrival at the laboratory, samples should be inspected and recorded to ensure compliance with a properly packed and transported sample, including ruling out sample leakages and contamination. Existing standard precaution and infection prevention and control (IPC) practices in hospitals, health clinics, and laboratories should be followed while collecting and handling specimens. At the testing laboratory, specimen volume, colour, and appearance should be documented (*e.g.*, yellow and cloudy, dark yellow and hazy, pale yellow and clear).

4. Sample Collection Forms

Complete a sample collection form for each sample to capture descriptive information to help correctly establish drivers of community-acquired UTI caused by AMR pathogens. An example of the sample collection is attached (**Annex 1**). Implementing a unique sample identification numbering system between all surveillance laboratories is important so that every sample, regardless of origin, has a unique sample identification.

5. SOPs, Training, and Trialing Sample Collection

Once the sampling plan is in place, preparing a detailed standard operating procedure (SOP) will ensure that sampling and laboratory practices are standardized across sites. Necessary SOPs to implement this protocol include but are not limited to IPC practices, Selection of UTI cases at health facilities, Collection of samples, sample management (handling, storage, and preparation for transport), Sample transportation and tracking, and Laboratory procedures.

Once SOPs have been prepared, staff at health facilities should be trained on those that pertain to their roles. Training should include active participation and practice, sampling, and form completion. Following this training, staff in each surveillance area should receive supportive supervision during an initial trial period to ensure that SOPs are followed correctly and receive additional on-site guidance about procedures.

6. Review the Sampling Plan

Although the sampling plan has been developed with the best information available, it is key to revisit the plan after implementation to ensure it achieves the aims of the surveillance activities and that involved stakeholders, health facilities, and laboratories find the approach and methodology acceptable and feasible. Some points to consider during the review include: 1) Can the involved health facilities manage the number and frequency of samples being collected? 2) Is there clarity about who is responsible for collection, storage, and transport? 3) Can the involved laboratories manage the number and frequency of samples collected? 4) Is the quality of samples suitable for diagnostic testing? 5) Does the pattern of uropathogens reflect what was expected epidemiologically? If the pattern of uropathogens does not reflect what was expected, determine if the deviation could be due to sample

quality, reagent quality, sample processing quality, culture or identification methods, cross-contamination between samples during collection or processing, or recording errors.

4 **RESPONSIBILITY OF NATIONAL AMR REFERENCE AND REGIONAL/PROVINCIAL SURVEILLANCE LABORATORIES**

1. Regional/Provincial Surveillance Laboratories

Ideally, culture, identification, and AST for each bacterial isolate against the panel of antimicrobials should be conducted at the regional/provincial surveillance laboratory within each surveillance area. This enables samples to be processed more quickly and ensures that the AMR reference laboratory is not overwhelmed. However, if this is not possible due to technical capacity, regional/provincial surveillance laboratories should carry out whichever procedures are possible, with the AMR reference laboratory conducting those that remain. Detailed procedures for AST should be obtained from EUCAST or CLSI guidelines, as appropriate and agreed upon by the National AMR Surveillance Committee or equivalent.

2. AMR Reference Laboratory

The AMR Reference Laboratory shall conduct any procedures outlined in the protocol that cannot be conducted at the regional/provincial surveillance laboratories. This will vary by country, and if the capacities of regional/provincial laboratories are not known, a readiness assessment may be needed before protocol implementation. Furthermore, the AMR reference laboratory should ensure that staff at regional/provincial laboratories are appropriately trained, maintain established and documented standards, and conduct quality control and assurance. In addition, the reference laboratory can provide additional identification and AST using other methods for comparison.

5 **LABORATORY PROCEDURES**

The AMR reference laboratory should coordinate the development of SOPs for sample processing, bacterial identification, AST, reporting, storage, and transport. Local staff should trial these in regional/provincial laboratories and adjust according to feedback. SOPs should align with national and international guidelines.

1. Urine specimen culture

Urine culture should be carried out according to laboratory SOPs within a maximum of two hours of collection for unpreserved specimens, within 8 hours for preserved specimens or specimens in a cool box with icepacks, and within 24 hours if refrigerated after collection using a quantitative method.

Inoculum of a known volume of a urine sample (e.g., 10µl, 5µl, 2µl, 1µl, etc.) should be spread on selected culture media plates as per SOP. A testing laboratory must prepare an SOP for Urine Culture per the attached example (Supplemental File 2). Inoculated plates need to be incubated at 35 ± 2°C for 16-24 hours and read for growth. The number of colonies will be multiplied by the appropriate factor to give the colony count per mL of urine. Bacterial counts of $>10^4$cfu/mL of no more than two species of microorganisms will indicate UTI (Table 4) [4]. Contamination should be defined as $> 10^4$ growth of more than two species or any growth of $< 10^4$ for MSU [4].

Table 4: Example of number colonies and interpretation

A	B	C	D
**Corresponding CFU/ml**	**Number of colonies**		
	**1µl**	**5µl**	**10µl**
10Λ4	10	50	100
10Λ5	100	500	1000

2. Urinalysis

Depending on the laboratory SOPs for processing urine for culture, the urinalysis may be done by dipping/completely immersing the strip into the urine sample following the laboratory's standard SOP for urinalysis at the collection site or the testing laboratory [5]. The urinalysis results are critical in some laboratories when deciding whether samples will proceed for culture. Boric acid may inhibit leukocyte esterase dipstick readings; dipstick testing performed on a sample in a boric acid container should be interpreted with caution.

3. Identification of Isolates

Depending on the level of the laboratory processing the samples, bacteria can be identified using a conventional method where multiple tests are performed, connoting the fermentation abilities, presence of certain enzymes, and certain biochemical reactions. For example, Enterobacterales can be recovered from culture using different biochemical tests, which should be described in standard operating procedures**.** In addition, in advanced, well-equipped laboratories, semi-automated techniques such as Matrix-Assisted Laser Desorption/Ionization-Time of Flight (*MALDI-TOF*) mass spectrometry (MS), Vitek 2, BD Phoenix, and Microscan can be used to identify bacteria accurately.

4. Antimicrobial Susceptibility Testing

Depending on the level of laboratory processing of the samples, AST can be performed usingthods such as the Kirby–Bauer disk diffusion method or semi-automated techniques such as VITEK-2, BD Phoenix, and the conventional me Microscan system. The testing laboratory should have an Antimicrobial Susceptibility Testing SOP based on the available method. MDR will be defined as acquired non-susceptibility to at least one agent in three or more antimicrobial categories [6].

4.1 Screening and Confirmation of ESBL

ESBL-producing isolates should be screened and confirmed as EUCAST/CLSI guidelines recommend [7]. The testing laboratory should have an ESBL Screening and Confirmation SOP.

5. Isolate Storage

Isolates should be safely transported to the AMR Reference laboratory monthly for additional tests and storage at -80 ^{O}C. The stored isolates may be used for other studies on AMR, including, but not limited to, molecular analysis.

6 **PROFICIENCY TESTING AND USE OF ATCC STRAINS FOR INTERNAL QUALITY ASSURANCE**

1. Proficiency testing

The national AMR reference and surveillance laboratories should have a well-established system per available guidelines for proficiency testing to ensure reliable diagnostic results are produced in laboratories. Proficiency testing should start after training in bacterial

culture, identification, and AST has been completed and once the laboratories have received quality reagents and consumables. The suggested steps towards proficiency testing are described as follows:

1) The AMR reference laboratory must ensure that it can produce repeatable AST results by testing each target bacteria against all targeted antibiotics for a particular bacterium as per CLSI/EUCAST guidelines. The laboratory should ensure it can produce repeatable results before developing the proficiency testing panel to send to the surveillance/satellite laboratories.

2) Initially, the AMR reference laboratory should send a panel of known isolates of the target bacteria relevant to each surveillance laboratory, with their identity and resistance profile disclosed, and request the laboratory to test each against the full panel of antibiotics listed for each isolate. This will enable the surveillance laboratories to test that they can identify the bacteria and achieve the known AST results. A standard form should be sent with each panel of isolates for the surveillance laboratory to complete the AST results and ensure standardisation in reporting results across all laboratories.

3) Subsequently, the AMR reference laboratory should send blinded samples, i.e., an isolate with identity and resistance undisclosed, with the culture request. The laboratory should identify the bacteria and test them against the appropriate panel of antibiotics.

4) Surveillance laboratories should report the results to the reference laboratory. If the results are correct, sampling may proceed. If the results are incorrect, the reference laboratory should review them and recommend corrective actions before repeating the proficiency testing.

5) This process may be repeated quarterly in the first year to ensure the laboratories perform reliably and offer accurate results. Subsequently, proficiency testing may be conducted at longer intervals.

2. Use of ATCC strains for Internal Quality Assurance

Each laboratory participating in AMR surveillance should have standard ATCC strains for Internal Quality Control. The ATCC strains should be tested once a week and for every new media batch. Each batch of media should be tested for sterility and performance. The zone diameter should be recorded for each ATCC strain each time it is tested. This information should be examined for consistency. Any issues identified in testing reliability should be investigated and rectified before further testing is conducted for the AMR surveillance program. AMR reference laboratories should participate in an External Quality Assurance (EQA) scheme from accredited EQA providers to ensure they are producing reliable results. To maintain the desired quality with the integrity of the culture stock, it is recommended that a bacterial culture should never be sub-cultured more than five times. The best way is to make aliquots of the ATCC strains when they are first received and to sub-culture each aliquot a maximum of five times, then autoclave and discard it.

7 **DATA MANAGEMENT, ANALYSIS, AND SHARING**

Collected data should be stored in secured cabinets or on a secure server that will be accessed by authorized personnel only. Utilize the WHONET and/or another data system to analyze as per standard operating procedures according to the stated objectives. WHONET software can generate standard reports to be shared within and across sectors, regions,

> and the world through global platforms such as the Global Antimicrobial Resistance Surveillance System (GLASS).
> Existing country regulations on data handling will govern data management. Collected data should be submitted to the AMR reference laboratory coordinating AMR surveillance in the country. The AMR reference laboratory conducts quality checks and data analysis, generates official AMR reports, and provides long-term data storage.

Protocol references

1. Baron EJ, Miller JM, Weinstein MP, Richter SS, Gilligan PH, Thomson Jr RB, et al. A guide to utilization of the microbiology laboratory for diagnosis of infectious diseases: 2013 recommendations by the Infectious Diseases Society of America (IDSA) and the American Society for Microbiology (ASM) a. Clinical infectious diseases. 2013;57(4):e22-e121.
2. Warren JW, Abrutyn E, Hebel JR, Johnson JR, Schaeffer AJ, Stamm WE. Guidelines for antimicrobial treatment of uncomplicated acute bacterial cystitis and acute pyelonephritis in women. Clinical Infectious Diseases. 1999;29(4):745-59.
3. Organization WH. Prioritization of pathogens to guide discovery, research and development of new antibiotics for drug-resistant bacterial infections, including tuberculosis. World Health Organization, 2017 9240026436.
4. Hay AD, Birnie K, Busby J, Delaney B, Downing H, Dudley J, et al. The Diagnosis of Urinary Tract infection in Young children (DUTY): a diagnostic prospective observational study to derive and validate a clinical algorithm for the diagnosis of urinary tract infection in children presenting to primary care with an acute illness. Health technology assessment. 2016;20(51).
5. Kupelian AS, Horsley H, Khasriya R, Amussah RT, Badiani R, Courtney AM, et al. Discrediting microscopic pyuria and leucocyte esterase as diagnostic surrogates for infection in patients with lower urinary tract symptoms: results from a clinical and laboratory evaluation. BJU international. 2013;112(2):231-8.
6. Magiorakos A-P, Srinivasan A, Carey RB, Carmeli Y, Falagas M, Giske CG, et al. Multidrug-resistant, extensively drug-resistant and pandrug-resistant bacteria: an international expert proposal for interim standard definitions for acquired resistance. Clinical microbiology and infection. 2012;18(3):268-81.
7. Poulou A, Grivakou E, Vrioni G, Koumaki V, Pittaras T, Pournaras S, Tsakris A. Modified CLSI extended-spectrum β-lactamase (ESBL) confirmatory test for phenotypic detection of ESBLs among Enterobacteriaceae producing various β-lactamases. Journal of clinical microbiology. 2014;52(5):1483-9.
