# Supplementary material for: Protocol for surveillance of antimicrobial-resistant bacteria causing community-acquired urinary tract infections in low-income countries
Source: PLoS One. 2024 May 31;19(5):e0304388. doi: 10.1371/journal.pone.0304388 (PMC11142477; doi:10.1371/journal.pone.0304388)
Supplement: Attachment — Submitted filename: AMR REVIEW.docx [file pone.0304388.s002.docx]

What do you mean by lower health facility?

Is 12 months data enough to do seasonal trend? What analytical techniques will be used on a 12 months data for seasonal trends. Is seasonal trend and seasonal variation the same? How do you define seasonal trend?

What is WHO?? You don’t start a sentence wit abbreviation. Understand that WHO is a standard abbreviation but it wasn’t defined prior to it’s use.

“In most LMIC settings, UTI is empirically treated with no data to support the choice of antibiotics, hence contributing to resistance development. Continuous AMR data on community-acquired pathogens are needed to draft empirical treatment guidelines, especially for areas with limited culture and susceptibility testing”…………………..**REFERENCE??**

“With such data, policymakers can design appropriate AMR control strategies that help guide community practitioners. Surveillance data on UTI can assist in understanding community-level magnitude, drivers, and transmission dynamics.”…….Have there been documented evidence that support this assertion? **Please reference them!!**

“Despite the importance of addressing antibiotic-resistant pathogens in the community setting,

protocols for the surveillance of AMR bacterial infections are lacking in most LMICs.”…….**REFERENCE?**

“Access to data about AMR drivers in community settings is necessary for governments and scientists to make evidence-based policy and intervention decisions. The protocol is required to assist users in designing a sustainable surveillance program for AMR and Community-Acquired

Infections (CAI).”…… Are these based on assumptions or there are documented evidence to support this standpoint? Kindly reference them!!!

“AMR surveillance should be ongoing to track new and existing resistance patterns and inform policy and decision-making about how to mitigate the consequences of AMR identified.”….. **NOT CLEAR!**
