# Supplementary material for: Protocol for surveillance of antimicrobial-resistant bacteria causing community-acquired urinary tract infections in low-income countries
Source: PLoS One. 2024 May 31;19(5):e0304388. doi: 10.1371/journal.pone.0304388 (PMC11142477; doi:10.1371/journal.pone.0304388)
Supplement: Attachment — Submitted filename: Response to Reviewers.docx [file pone.0304388.s003.docx]

**POINT TO POINT RESPONSE TO EDITOR AND REVIEWERS’ COMMENTS**

**Editor’s comments**

1. Please ensure that your manuscript meets PLOS ONE's style requirements, including those for file naming.

**Response:** We have checked and confirmed that the manuscript meets the PLOS ONE style requirement

2. We note that the grant information you provided in the ‘Funding Information’ and ‘Financial Disclosure’ sections do not match.

**Response:** We have corrected grant information in the funding information and financial disclosure section. The amended statement is included in the cover letter

3. Thank you for stating the following in the Acknowledgments Section of your manuscript:

"We thank the Ending Pandemics program, Connecting Organizations for Regional Disease Surveillance (CORDS), and East and Southern Africa regional Technical Working Group members for providing valuable insight. We are grateful for the guidance provided by the Ending Pandemics Team. We gratefully acknowledge the technical and funding support received for developing this protocol from the Fleming Fund Grant provided by the UK Department of Health and Social Care (DHSC) and the Mott MacDonald agent for administering this grant."

Please note that funding information should not appear in the Acknowledgments section or other areas of your manuscript. We will only publish funding information present in the Funding Statement section of the online submission form.

Please remove any funding-related text from the manuscript and let us know how you would like to update your Funding Statement. Currently, your Funding Statement reads as follows: "The Fleming Fund Regional grant number FF49/440, Regional Grants Round 2, funded the development of this protocol. The funder had no involvement in the design and development of the protocol." Please include your amended statements within your cover letter; we will change the online submission form on your behalf.

**Response:** We have removed the funding-related text from the acknowledgement and other areas of the manuscript. We have rephrased the funding statement and included the amended statement in the cover letter.

4. When you submit your revision, please provide a PDF version of your protocol as generated by [protocols.io](http://protocols.io)(the file will have the [protocols.io](http://protocols.io) logo in the upper right corner of the first page) as a Supporting Information file. The filename should be S1_file.pdf, and you should enter “S1 File” into the Description field. Any additional protocols should be numbered S2, S3, and so on.

If you have not yet uploaded your protocol to [protocols.io](http://protocols.io), you are invited to use the platform’s protocol entry service [<https://www.protocols.io/we-enter-protocols>] for doing so, at no charge. Through this service, the team at [protocols.io](http://protocols.io) will enter your protocol for you and format it in a way that takes advantage of the platform’s features. When submitting your protocol to the protocol entry service please include the customer code PLOS2022 in the Note field and indicate that your protocol is associated with a PLOS ONE Lab Protocol Submission. You should also include the title and manuscript number of your PLOS ONE submission.

**Response:** We uploaded the protocol to protocols.io and provided the PDF version generated by protocols.oi as a supporting information file (S1_file).

**Reviewers' comments:**

**Reviewer #1:**

What do you mean by lower health facility?

**Response:** we have replaced the lower health facility with “primary health facility” throughout for clarity.

Is 12 months data enough to do seasonal trend? What analytical techniques will be used on a 12 months data for seasonal trends. Is seasonal trend and seasonal variation the same? How do you define seasonal trend?

**Response:** Thanks for the comment. We propose 12 months to observe if there are recurring patterns or fluctuations in AMR data for 12 months. The 12-month data may show time series variation influenced by weather and other calendar-related events. We have rephrased the statement. Line 41-42

What is WHO?? You don’t start a sentence wit abbreviation. Understand that WHO is a standard abbreviation but it wasn’t defined prior to it’s use.

**Response:** We have corrected this by writing the long form before the abbreviation. Line 64-65

“In most LMIC settings, UTI is empirically treated with no data to support the choice of antibiotics, hence contributing to resistance development. Continuous AMR data on community-acquired pathogens are needed to draft empirical treatment guidelines, especially for areas with limited culture and susceptibility testing” - **REFERENCE??**

**Response:** We have added reference # 11, line 71

“With such data, policymakers can design appropriate AMR control strategies that help guide community practitioners. Surveillance data on UTI can assist in understanding community-level magnitude, drivers, and transmission dynamics. Have there been documented evidence that support this assertion? **Please reference them!!**

**Response:** We have added a reference # 12, line 72

“Despite the importance of addressing antibiotic-resistant pathogens in the community setting, protocols for the surveillance of AMR bacterial infections are lacking in most LMICs.” **REFERENCE?**

**Response:** We have added two references #13 and 14, line 76

“Access to data about AMR drivers in community settings is necessary for governments and scientists to make evidence-based policy and intervention decisions. The protocol is required to assist users in designing a sustainable surveillance program for AMR and Community-Acquired Infections (CAI). Are these based on assumptions or there are documented evidence to support this standpoint? Kindly reference them!!!

**Response:** We have added a reference # 12, Line 80

“AMR surveillance should be ongoing to track new and existing resistance patterns and inform policy and decision-making about how to mitigate the consequences of AMR identified. **NOT CLEAR!**

**Response:** We have rephrased the paragraph for clarity. Line 105-108

**Reviewer 2**

1. WHO has classified Enterobacterales carbapenem-resistant and 3rd generation cephalosporin-resistant as critical priority pathogens – This statement is unclear

**Response:** we have rephrased the statement for clarity. Line 64-67

2. who are residents of a given surveillance area and passively presenting to a health facility for health care – please clarify what ‘passively’ means in this case

**Response:** We have removed the word passively in the protocol and revised the statement in the manuscript. Line 37-38

3. In addition, the protocol aligns with WHO AMR surveillance guidelines – Reference required

**Response:** We have added a reference # 17, line 89

4. community-acquired urinary tract infections in low-income countries are published on [protocols.io](http://protocols.io). – Do the writers mean low income or low-middle income?

**Response:** We have corrected the statement to low-middle income. Line 90

5. Table 1 – Lab tests such as urinalysis and urine microscopy are not usually regarded as ‘signs’

**Response:** we have renamed the column in Table 1 of the protocol as clinical description instead of symptoms and Signs. S1, page 4

6. Table 3 – May be rather simplistic as it does not into account intrinsic resistance

**Response:** We indicated in the text that antimicrobial agents for AST should be selected from the critically important antimicrobial classes based on WHO recommendations and used to treat UTI. We have revised the text related to Table 3 of the protocol to indicate that the list of antibiotics is provided as an example. S1, Page 5

7. For urinalysis- Include any caution in a specimen preserved with boric acid

**Response:** The caution in a specimen preserved with boric acid has been included in the protocol. S1, paragraph 1, page 9.

8. Isolates should be safely transported monthly to the AMR Reference laboratory for additional tests and storage at -80 OC per country SOP for isolate management – is storage at this temperature possible at district level?

**Response:** Storage at minus 80 OC is impossible at the district level. We were referring to storage at a reference laboratory. For clarity, we have rephrased the statement in the protocol. S1, Page 9

9. The QA for AST does not comply with CLSI as stated in the protocol

**Response:** We have revised the statement for QA for AST that contradicted the CLSI guideline. SI, Paragraph 1, page 10

Overall, a more comprehensive protocol will allow for seamless implementation.

**Response:** After addressing the reviewers’ comments, the protocol looks comprehensive for smooth implementation of AMR surveillance
